# *BACH2* promotes indolent clinical presentation in Waldenström macroglobulinemia

**DOI:** 10.18632/oncotarget.9917

**Published:** 2016-06-07

**Authors:** Charles Herbaux, Elisabeth Bertrand, Guillemette Marot, Christophe Roumier, Nicolas Poret, Valérie Soenen, Olivier Nibourel, Catherine Roche-Lestienne, Natacha Broucqsault, Sylvie Galiègue-Zouitina, Eileen M Boyle, Guillemette Fouquet, Aline Renneville, Sabine Tricot, Franck Morschhauser, Claude Preudhomme, Bruno Quesnel, Stephanie Poulain, Xavier Leleu

**Affiliations:** ^1^ Inserm U837, Team 3, Cancer Research Institute of Lille, Lille, France; ^2^ Service des Maladies du Sang, Hôpital Huriez, CHRU, Lille, France; ^3^ Lille Nord de France University, Equipe Biostatistique, UDSL, Lille, France; ^4^ Laboratory d’Hématologie, Biologie and Pathologie Center, CHRU, Lille, France; ^5^ Département d’Hématologie-Immunologie-Cytogénétique, CH, Valenciennes, France; ^6^ Service d’Hématolgie et Thérapie cellulaire, Hématologie, CHU, Poitiers, France; ^7^ Centre d’Investigation Clinique Inserm, CHU, Poitiers, France

**Keywords:** BACH2, Waldenström macroglobulinemia, indolent, progression, physiopathology

## Abstract

Approximately 30% of the patients who fulfil the criteria of Waldenström's macroglobulinemia (WM) are diagnosed while asymptomatic (indolent), and will not require immediate therapy. Conversely, patients with a disease-related event will be considered for therapy. The physiopathology of these 2 groups remains unclear, and the mechanisms of progression from indolent to symptomatic WM have yet to be fully understood. Seventeen patients diagnosed with WM were included in this study, 8 asymptomatic WM (A-WM) and 9 symptomatic WM (S-WM). A differential analysis was performed on a first series of 11 patients and identified 48 genes whose expression separated samples from A- to S-WM. This gene signature was then confirmed on a second independent validation set of 6 WM. Within this expression profile, *BACH2*, a B-cell transcription factor known to be a tumor suppressor gene, was found to be over-expressed in A-MW relatively to S-MW. We specifically over-expressed *BACH2* in a WM-related cell line and observed a significant reduction of the clonogenic activity. To the best of our knowledge, we report for the first time a specific gene expression signature that differentiates A-WM and S-WM. Within this expression profile, BACH2 was identified as a candidate gene that may help to understand better the behavior of tumor cells in indolent WM.

## INTRODUCTION

Waldenström's macroglobulinemia (WM), a rare lymphoproliferative disorder characterized by bone marrow (BM) infiltration of lymphoplasmacytic cells associated with serum IgM paraprotein [1], has various clinical presentations. Mainly, WM is characterized with either symptomatic (S-WM) or asymptomatic (A-WM also called smoldering or indolent WM) features, according to the presence or absence of features attributable to tumor infiltration and/or attributable to the monoclonal protein [2]. The exact incidence rate of A-WM is unknown as many patients are possibly undiagnosed, and one might consider it represents the majority of WM [3], but the consequence for the patients is remarkable as A-WM will not require immediate therapy sometimes for years. Conversely, patients with WM disease-related events will be considered for therapy [4].

The long lasting indolent profile is not specific to WM, and is observed virtually in all B-cell non Hodgkin low grade lymphoma subtypes. Understanding the key physiopathological mechanisms that protect tumor cells from progression towards aggressive/symptomatic disease might be a target for therapy in the very near future. Although progress was made in recent years to understand the pathogenesis of WM, very little is known about differences between A-WM and S-WM, and the mechanisms of progression from A-WM to S-WM have yet to be understood. Gene expression profiling has been shown to identify specific signature allowing clinical aggressiveness stratification in myeloma [5, 6] and in follicular lymphoma [7].

We therefore hypothesized that a gene signature that differentiates S-WM from A-WM could be identified. The purpose of this study was to find pathways and genes from this gene signature that will help better understand the underlying mechanisms that differentiate S-WM from A-WM.

## RESULTS

### A-WM and S-WM display different gene expression profile-based signatures

**Figure 1:**
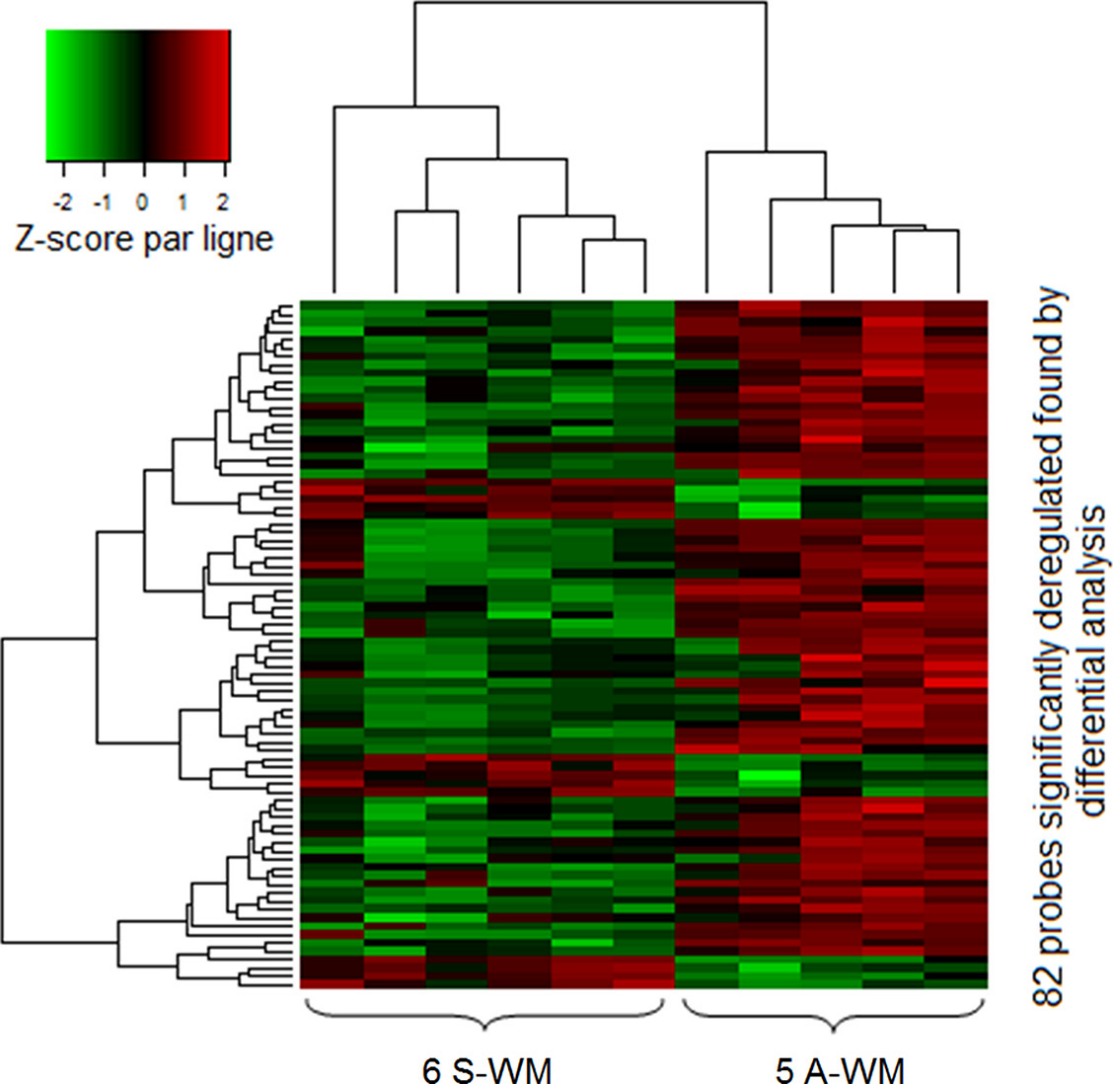
GEP identifies a signature that allows to differentiate A-WM from S-WM Gene expression data were normalized using the Robust Multi-array Average (RMA) algorithm. Then we performed differential analysis on a first series of 11 patients; 82 probes (corresponding to 48 genes) were significantly identified. The heatmap shows the spectrum of normalized expression values with a Z-score: red indicates a high level of mRNA expression and green indicates a low level of expression. Each row represents one of the 82 probes and each column a WM sample. In this unsupervised classification, patients with A-WM clustered separately from patients with S-WM.

We first studied a set of 11 patients (5 A-WM and 6 S-WM) with a high-throughput assay-based comprehensive approach. The differential analysis identified 82 probes corresponding to 48 genes whose patterns of expression resulting from using an unsupervised classification were able to differentiate samples from A-WM and S-WM (Figure 1) The 48 genes are listed in Supplementary Data Table 1.

**Table 1:** Characteristics of the patients which samples were used in this study

Patient	Age* / sex	MYD88 mutation	Clinical status	Criteria of symptomatic disease	M-comp at time of GEP (g/L)	GEP profile	6q deletion / *BACH2*deletion^†^
3	63/F	+	A-MW	NA	30	A	no / no
5	76/F	+	A-MW	NA	11	A	yes / yes
7	83/M	+	A-MW	NA	37	A (ivs)	uk
9	61/M	+	A-MW	NA	15	A	yes / no
25	63/F	−	A-MW	NA	26	A (ivs)	yes / no
28	63/F	+	A-MW	NA	19	A	no / no
30	68/F	−	A-MW	NA	6	A (ivs)	no / no
31	64/M	+	A-MW	NA	20	A	yes / no
1	81/M	+	S-MW	Autoimmune hemolytic anemia	26	S (ivs)	no / no
2	70/F	+	S-MW	Central anemia	37	S	yes / yes
4	69/M	+	S-MW	Central anemia	52	S	yes / yes
6	62/F	−	S-MW	Central anemia	49	S	yes / no
8	73/F	+	S-MW	Central anemia	50	A (ivs)	uk
12	86/M	+	S-MW	Adenopathies	23	S	no / no
18	66/M	+	S-MW	Central anemia and cryoglobulinemia	19	S (ivs)	uk
20	65/M	+	S-MW	Pancytopenia	12	S	no / no
27	78/M	+	S-MW	Central anemia	25	S	yes / yes

Abbreviations: M: male, F: female, A-WM: asymptomatic WM, S-WM: symptomatic WM, ivs: independent validation set, uk: unknown, M-comp: monoclonal-component.

*At the time of diagnosis; †: by SNP.

### Validation of the 48 genes-based signature on an independent set of patients

**Figure 2:**
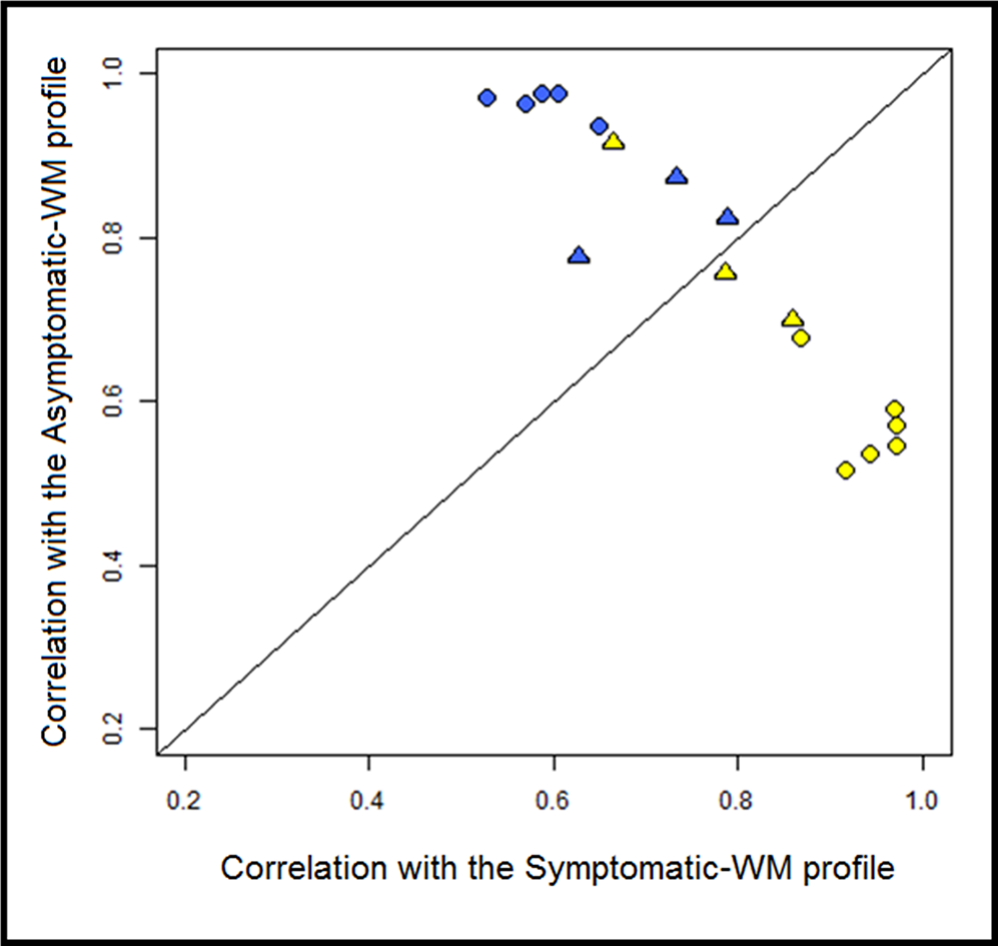
Correlation plot of the transcriptional profile for A-WM and S-WM The correlation coefficients for each of the WM samples with the average expression of the 82 probes in either A-WM or S-WM samples were calculated and are shown on the vertical axis and horizontal axis, respectively. The line of equal correlation was chosen as threshold. Patients positioned above the threshold were classified as A-WM; patients below the threshold were classified as S-WM. The blue dots correspond to the A-WM, the yellow ones to the S-WM. The circles correspond to the first 11 patients and the triangles to the 6 patients from the independent set.

We sought to validate the 48 genes-based signature obtained with the first set of 11 patients on an independent set of 6 patients with WM. For this supervised classification the objective was to study whether the A-WM and S-WM would be correctly identified and categorized using the 48 genes-based signature profile. Our gene expression-profiling-based signature was overall able to classify accurately 94% of the 17 WM patients, including the 6 WM of an independent validation set (Figure 2).

### Pathway analysis of in the signature

**Figure 3:**
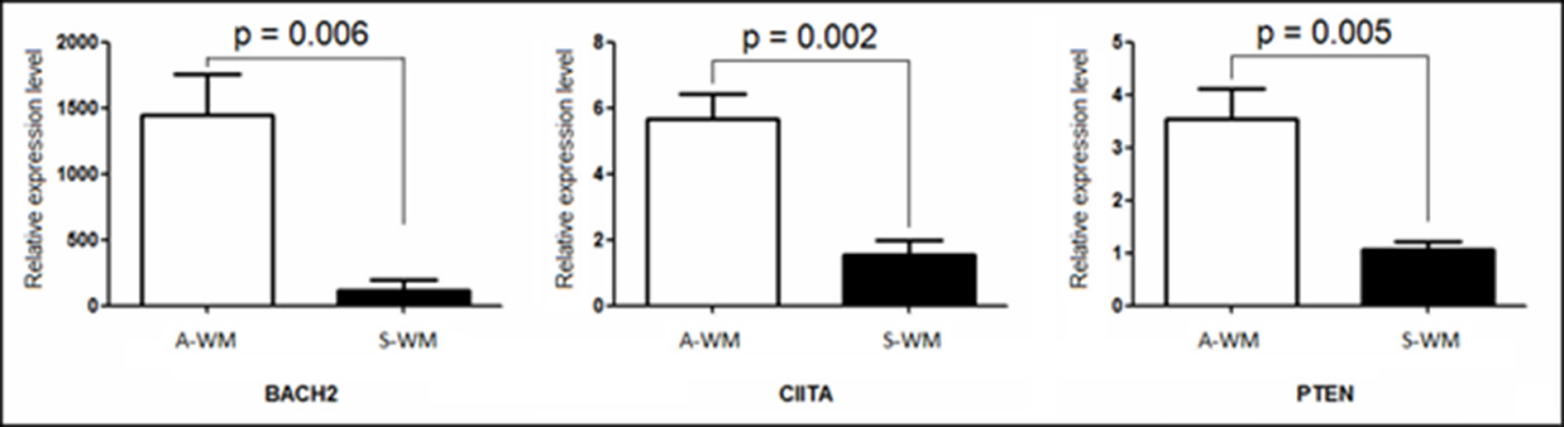
Confirmation of the gene expression profile by RT-qPCR Quantification of mRNA of *BACH2*, *CIITA* and *PTEN* was performed by real time qPCR against an endogenous control gene: ABL1. Histograms show relative expression values between A-WM and S-WM. The white histograms correspond to Asymptomatic WM, the black ones to Symptomatic WM. A statistically significant difference was observed on the three genes.

We then reviewed the literature and sought for specific pathways involved in the difference between A-WM and S-WM using Ingenuity^®^ software, on the 48 genes independently of the fold-changes. The pathway analysis identified three networks: the B cell differentiation and activation pathway, the *PI3K/AKT* pathway and the microenvironment interactions pathways (Supplementary Data Table 2). Following the review of the literature, 3 genes appeared of key interest: *BACH2*, *CIITA* and *PTEN*. *BACH2* is a B-cell-specific transcription factor known to be a tumor suppressor gene [8]. *CIITA* acts as a positive regulator of class II major histocompatibility complex gene transcription [9], *BACH2* increases the expression of *CIITA* through *PRDM1*. *PTEN* functions as a tumor suppressor by negatively regulating *AKT1* signaling, a pathway involved in WM [10]. We then sought to confirm the transcriptional dysregulation of these 3 genes on patients with WM, 3 A-WM and 4 S-WM, by qRT-PCR (Figure 3).

### Genomic alteration of *BACH2*

**Figure 4:**
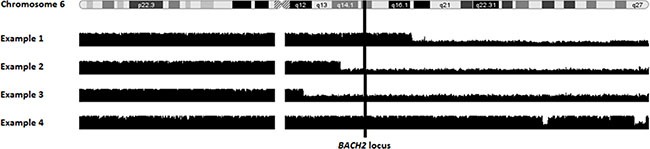
BACH2 locus alteration differs in patients with 6q deletion according to WM status Smooth signal of copy number variation is shown for 4 patients with a 6q deletion (del6q). The vertical line shows the *BACH2* gene location in 6q15 region. Examples 1 and 4 are asymptomatic patients, with a del6q preserving the 6q15 locus. Examples 2 and 3 are symptomatic patients with a del6q involving 6q15 locus.

In order to study further the mechanisms of deregulation of *BACH2* in A-WM and S-WM, we conducted a genome wide SNP array study on 14 out of the 17 WM included in this study. *BACH2* gene is located on the 6q15 locus. Among those 14 patients, 8 (4 A-WM and 4 S-WM) demonstrated a deletion of long arm of chromosome 6 (del6q), the most frequent chromosomal abnormality in WM, seen in approximately 50% of WM. Interestingly, we noticed that 3 out of the 4 S-WM with del6q had the 6q15 locus altered, whereas 3 out of 4 of the A-WM had a del6q preserving the 6q15 region (Figure 4).

### *BACH2* involved in clonogenic activity in A-WM

**Figure 5:**
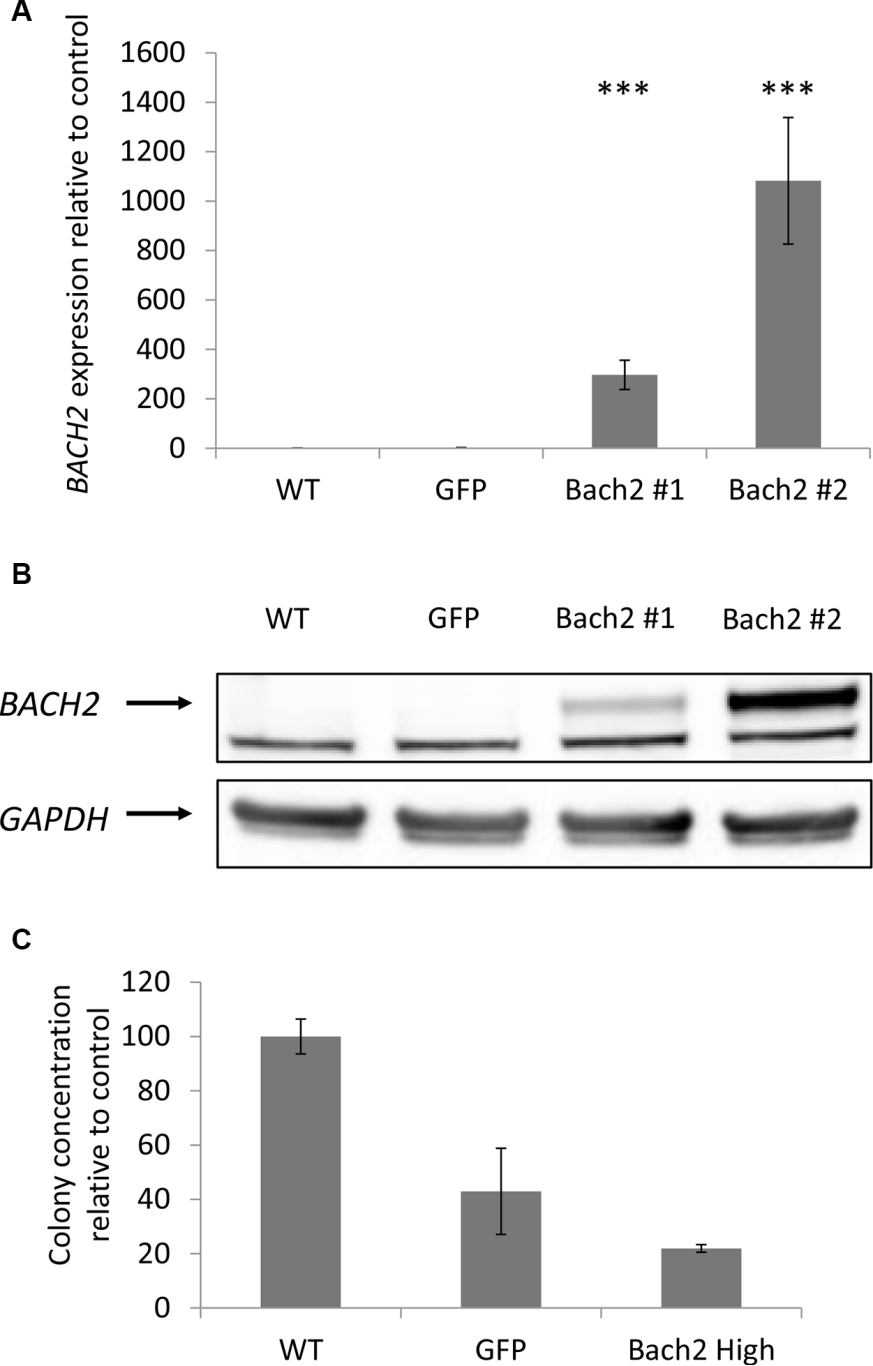
Clonogenic activity was significantly reduced in a *BACH2* high engineered cell line Expression of Bach2 in transfectants. (**A**) RNA level were measured by q-RTPCR in BCWM1 cell line non transduced (WT), infected with an empty particle (GFP) and in several transfectants (Bach2 #1 and #2). Histograms show relative expression values. (**B**) Protein expression of Bach2 was evaluated by Western blot in the same cell lines (*p* < 0,003). Only the highest Bach2 expressing clone was used for the next experiments. (**C**) Methylcellulose colony formation assay was performed on BCWM1 cell line as described in Materials and methods. As a control, BCWM1 non transduced (WT) and infected with an empty lentiviral particle (GFP) were used. Histograms show relative concentration values.

As mentioned above, we gave a special interest to *BACH2* following ingenuity pathway analysis and literature review. In our study, *BACH2* expression was increased in indolent WM relative to symptomatic WM, and thus we sought to reproduce *in vitro* the two asymptomatic and symptomatic WM profile through the study of the consequences of *BACH2* deregulation on a WM cell-line. Of interest, we noticed that BCWM1 was the most proliferative WM cell line (as compared to MWCL1 for example), *in vitro*. We first specifically over-expressed *BACH2* in BCWM1, as presented in panel A and B of Figure 5. We then studied viability and cell growth. We did not observe any difference between wild type BCWM1 and *BACH2* high engineered BCWM1 (data not shown). We then focused on studying the clonogenic activity according to the level of *BACH2* expression, another major phenomenon and key feature of tumorigenesis. As shown on Figure 5C, we noticed that the clonogenic activity was significantly reduced in *BACH2* high engineered cell lines.

## DISCUSSION

We have identified a specific gene expression signature of 48 genes that differentiates A-WM and S-WM, validated on an independent set of patients. Some genes appeared of key interest to explain the differences observed between A-WM and S-WM. *BACH2* is a B-cell transcription factor known to act as a tumor suppressor gene. *CIITA* is part of the same signaling pathway as *BACH2; BACH2* represses *BLIMP1* which represses *CIITA*. Otherwise, it was demonstrated that primary WM cells present a constitutive activation of the *PI3K/Akt* pathway [10], sustained by decreased expression of *PTEN*. In our study, *BACH2*, *CIITA* and *PTEN* expression is lower in S-WM than in A-WM. The suppressor of cytokine signalling-6 (*SOCS6*) is over-expressed in tumor cells of patients with symptomatic WM. In this particular case, two pathophysiological hypotheses are possible. First, SOCS6 could inhibit antitumor immunity by suppressing effects of IFN-alpha, as described for *SOCS3* in T lymphoma [11]. On the other hand, the over-expression of *SOCS6* could be an epiphenomenon, constituting a negative feedback loop in response to multiple cytokine stimulation experienced by aggressive WM tumor cells, as described for *SOCS1* in chronic myeloproliferative disorders [12]. Other genes could have also been interesting candidates considering their important fold-change and low *p*-value in the differential analysis, the list is available in Supplementary Table 1.

One of the important findings of this study was the identification of *BACH2* as a potential key player in the aggressiveness of WM tumor cells. *BACH2* is a transcription factor that is selectively expressed in B-cells, abundantly expressed in B-cell early stages and turned off in terminally differentiated cells [13]. This protein has multifaceted actions in B cell development [8], from tumor suppressor gene to class switch recombination and somatic hypermutation [14]. Most importantly, *BACH2*-mediated activation of TP53 is required for elimination of pre-B cells that failed to productively rearrange immunoglobulin gene [15]. Many studies have examined the role of *BACH2* in lymphoid neoplasms in relation to its critical actions in the B lymphocyte development and ontogeny, particularly as a tumor suppressor gene [13, 16, 17]. Importantly, it was shown that *BACH2* reduced proliferation and induced cell death when over-expressed in B lymphoma tumor cells [17], and enforced expression of *BACH2* in RAJI cell line resulted in marked reduction of clonogenic activity [8]. These findings are consistent with the key functions of *BACH2* in the lymphomagenesis, in DLBCL, but also apparently in WM as we have shown, although the exact mechanism of action of *BACH2* remains to be identified. *BACH2* was previously mentioned in WM in two studies [18, 19], as part of a gene expression profile. The first study discriminated clonal B lymphocytes from WM and CLL, while the second one discriminated Waldenström's clone and normal CD22+CD25− B-cells. Interestingly, the second study showed that *BACH2* was under-expressed in Waldenström's clone (clonal B-cells from IgM MGUS and WM patients), consistent with our findings, and provides additional argument for the assumption that *BACH2* plays a tumor suppressor gene-role in WM.

It was described that the frequency of WM patients displaying genomic alteration (like copy-number alterations or loss of heterozygosity) significantly increased from asymptomatic to symptomatic WM [19, 20]. It is therefore expected that these genomic abnormalities might cause a significant modification of gene expression profile, as shown in our study for the first time to the best of our knowledge. Surprisingly, Paiva *et al*. [19] reported absence of difference between the gene expression profile of A-WM versus S-WM; possibly explained by the lower number of patients in their transcriptomic study (4 asymptomatic and 6 symptomatic). Furthermore, Paiva *et al*. used FACS-sorted cells, while we used immunomagnetic beads sorted cells (purity of sample was then confirmed by flow cytometry).

We showed that the MDR (minimal deleted region) of del6q seemed to differ in size for A-WM and S-WM, and also seemed to differ in the integration of the 6q15 locus, where *BACH2* is located. Indeed, among patients with del6q, we noticed that patients with S-WM demonstrated more frequently a del6q involving the 6q15 locus. We therefore concluded that haploinsufficiency could participate in the under-expression of *BACH2* in symptomatic WM

In our study, *BACH2* expression did not seem to be correlated with viability and cell growth in WM, but only with clonogenic activity. The same data was reported in RAJI cell line [8]. In this study, the authors also performed two kinds of assays. One was to monitor growth curves, and the other was to monitor colony formation activity. The latter assay is thought to be more stringent in terms of cell proliferation.

Finally, these data might have therapeutic applications. Targeting a tumor suppressor gene is known to be difficult, but the *BACH2* pathway could be targeted. For example, *BACH2* represses *BLIMP1*. A *BLIMP1* inhibitor could possibly guide symptomatic WM cells in a more indolent phenotype.

We report for the first time a GEP signature allowing to distinguish asymptomatic from symptomatic WM. Using this set of data we have identified key pathways and genes to help further understand the mechanisms of progression and the physiopathology of WM. In particular, *BACH2* expression seems to be significantly correlated with the phenotypic differences between asymptomatic and symptomatic WM. Future studies will help validate this gene signature and might decipher the role of *BACH2* in WM.

## MATERIALS AND METHODS

### Patients

Seventeen patients diagnosed with WM [1] (8 A-WM and 9 S-WM) were included in this study. Table 1 summarizes the main characteristics of the patients. Patients were untreated and gave informed consent prior to research sampling. The median age was 70 years (range, 61–86). Approval of this protocol was obtained from the local IRB of the CHRU of Lille (CSTMT043) and the study was conducted in accordance with the Declaration of Helsinki.

Conventional Cytogenetic, FISH, Immunophenotypic studies were available for all the patients and performed as previously published [20].

### Cell selection

Research sampling consisted of BM samples and blood cells for all WM patients. T lymphocytes normal fraction was used as an intra-individual reference to distinguish acquired somatic aberrations in WM from germline polymorphisms. The samples were enriched for cell population of interest using immunomagnetic beads (B cell isolation kit for tumoral cells, and Pan T cell isolation kit, Myltenyi-Biotec, USA). The purity of samples was confirmed by flow cytometry on tumor (CD19, kappa and lambda) and normal cell fractions (CD3, T lymphocytes). DNA was extracted using Qui Amp kit (Sigma-Aldrich Co. Saint-Quentin Fallavier France). Total RNA was extracted using the Trizol method (Invitrogen, Carlsbad CA).

### Cell line and lentiviral infection

BCWM.1 cell line was used in this study. This is a B-cell line developed in patients with WM; BCWM.1 was a gift from Dr S.P. Treon from DFCI (Dana-Farber Cancer Institute) [21], and harboring MYD88 L265P mutation but no 6q deletion. Lentiviral constructs expressing GFP and resistance to puromycin used to generate stable Bach2 expressing BCWM.1 cell line (BCWM.1-Bach2) and empty vector (BCWM.1-GFP) were purchased from Applied Biological Material (ABM) Inc. (Richmond, BC). BCWM.1 cell line was exposed to lentiviral particles for 6 hours at a multiplicity of infection (MOI) of 2. Two days after the infection, selection of the infected cells was done by adding puromycin to the culture medium at a concentration of 1 μg/mL.

### Proliferation, viability, immunoblotting and clonogenicity assay

Viability and cell growth were determined using the MTS assays, CellTiter 96^®^ AQueous One Solution Cell Proliferation Assay (Promega) and the Click-iT^®^ EdU Flow Cytometry Assay kit (Invitrogen) according to the manufacturer's protocol, respectively. Apoptosis was measured using annexin V - propidium iodide staining using flow cytometric analysis (Beckman Coulter, Brea, CA, USA). Study of mitochondrial membrane potential was achieved using MitoTracker Red CMXRos-Special Packaging^®^ (Invitrogen). Study of caspases activity was performed using staining CaspaLux^®^ (OncoImmunin, Gaithersburg, MD). The clonogenic activity of each BCWM1 clone was evaluated by methylcellulose culture. 500 cells were placed in 1 ml of methylcellulose medium without cytokines (MethoCult H4230, Stemcell technologies, Vancouver, Canada) in duplicates, and incubated under fully humidified conditions in 35 mm Petri dish (Greiner Bio-One, Kremsmünster, Austria) at 37°C in 5% CO2. Colonies were numbered on day 10. The following antibodies were used for immunoblotting on whole cell lysates: anti-BACH2 (LSBio^®^, Seattle, WA, USA) and anti-actin (Santa Cruz Biotechnology, Santa Cruz, CA, USA).

### Real-time quantitative reverse transcription PCR

Quantification of mRNA of BACH2, CIITA and PTEN was performed by real time qPCR against an endogenous control gene, ABL1, with correspondent Taqman^®^ assays on StepOneTM Real-Time PCR System (Life Technologies, CA, USA). Relative expression values were calculated using the means of Δ Ct (threshold cycle) from the triplicates and expressed as 2-DDCt.

### Single nucleotide polymorphism array analysis and gene expression profiling

Genome-wide detection of copy number alteration (CNV) and loss of heterozygosity (LOH) were available in 13 of the 17 WM cases using the Genome-Wide Human SNP Array 6.0 (Affymetrix) [20]. It allows precise delineation of the location and size of submicroscopic chromosomal defects, copy-neutral LOH and germline CNVs.

Gene expression profiling (GEP) was performed using U133 Plus 2.0 arrays (Affymetrix, USA), that analyzed over 47,000 transcripts. The RNA integrity was assessed using 2100 Bioanalyzer (Agilent, CA, USA). Expression data were normalized using the Robust Multi-array Average (RMA) algorithm. We performed differential analysis on a first series of 11 patients (5 A-WM and 6 S-WM) calculated with the ‘metaMA’ [22] R package (with the ‘limma’ option) in order to establish a gene expression profile. We then used a supervised classification to distinguish A-WM from S-WM as previously described [23]. Therewith, we validated this profile on an independent set of 6 patients (3 A-WM and 3 S-WM). We then performed a pathway analysis using Ingenuity Pathways Analysis^®^ (Ingenuity Systems, CA, USA).

### Other statistical analyses

Statistical associations were assessed using Chi2 and Fisher exact tests. Pairwise comparisons were performed by Mann-Whitney test or Student's *t*-test. Overall survival was defined as the time between diagnosis and death or the last follow-up. Statistics were achieved using SPSS 15.0.

## SUPPLEMENTARY MATERIALS TABLES




