# Supplementary material for: BACH2 promotes indolent clinical presentation in Waldenström macroglobulinemia
Source: Oncotarget. 2016 Jun 7;8(34):57451–9. doi: 10.18632/oncotarget.9917 (PMC5593656; doi:10.18632/oncotarget.9917)
Supplement: Supplementary file 1 [file oncotarget-08-57451-s001.pdf]

BACH2 promotes indolent clinical presentation in Waldenström macroglobulinemia

Supplementary Materials

Supplementary Data Table 1: 48 genes identified by the differential analysis See Supplementary Data Table 1

Supplementary Data Table 2: Deregulated genes in the three pathways identified by Ingenuity on the transpritional profile that differentiate A-WM from S-WM

Gene	Expression level between A-WM and S-WM
**B cells differentiation and activation pathway**	
BACH2	A-WM > S-WM
CIITA	A-WM > S-WM
RGS1	S-WM > A-WM
MMD	A-WM > S-WM
HLA-DOA	A-WM > S-WM
TLE4	S-WM > A-WM
PI3K/AKT **pathway**	
LYN	A-WM > S-WM
AKT3	A-WM > S-WM
PTEN	A-WM > S-WM
**Microenvironment interactions pathways**	
VCL	A-WM > S-WM
SOCS6	S-WM > A-WM
NEDD4L	A-WM > S-WM
FAIM3	A-WM > S-WM
FCRL1	A-WM > S-WM

A-WM > S-WM means that this gene is more expressed in patients with asymptomatic WM than in those with symptomatic WM. And conversely for S-WM > A-WM.
