# Supplementary material for: BACH2 promotes indolent clinical presentation in Waldenström macroglobulinemia
Source: Oncotarget. 2016 Jun 7;8(34):57451–9. doi: 10.18632/oncotarget.9917 (PMC5593656; doi:10.18632/oncotarget.9917)
Supplement: Supplementary file 2 [file oncotarget-08-57451-s002.doc]

**Table 1S:**

| **Official symbol** | **Official full name** | ***P-value*** | ***Fold- Change*** |
| --- | --- | --- | --- |
| *AKT3* | V-AKT MURINE THYMOMA VIRAL ONCOGENE HOMOLOG 3 | 4,01.10^-02^ | -1,34 |
| *ALOX5* | ARACHIDONATE 5-LIPOXYGENASE | 1,77.10^-02^ | -4,85 |
| *ASPH* | ASPARTATE BETA-HYDROXYLASE | 4,74.10^-03^ | -1,74 |
| *BACH2* | BTB AND CNC HOMOLOGY 2 | 5,88.10^-04^ | -4,99 |
| *CAT* | CATALASE | 4,53.10^-02^ | -1,10 |
| *CCDC109B* | COILED-COIL DOMAIN CONTAINING 109B | 2,87.10^-02^ | -1,89 |
| *CELSR1* | CADHERIN EGF LAG SEVEN-PASS G-TYPE RECEPTOR 1 | 4,65.10^-02^ | -1,41 |
| *CIITA* | MAJOR HISTOCOMPATIBILITY COMPLEX, CLASS II, TRANSACTIVATOR | 6,05.10^-03^ | -1,61 |
| *CLCN4* | CHLORIDE CHANNEL 4 | 1,46.10^-02^ | -2,77 |
| *ESYT1* | EXTENDED SYNAPTOTAGMIN-LIKE PROTEIN 1 | 4,01.10^-02^ | -1,24 |
| *EVL* | ENA/VASP-LIKE | 1,56.10^-02^ | -1,57 |
| *FAIM3* | FAS APOPTOTIC INHIBITORY MOLECULE 3 | 1,56.10^-02^ | -1,64 |
| *FAM49A* | FAMILY WITH SEQUENCE SIMILARITY 49, MEMBER A | 4,01.10^-02^ | 1,74 |
| *FCRL1* | Fc RECEPTOR-LIKE PROTEIN 1 | 4,01.10^-02^ | -1,27 |
| *GABBR1* | GAMMA-AMINOBUTYRIC ACID B RECEPTOR 1 | 4,11.10^-02^ | -1,39 |
| *GLIPR2* | GLI PATHOGENESIS-RELATED 2 | 2,87.10^-02^ | -1,01 |
| *GNG2* | GUANINE NUCLEOTIDE-BINDING PROTEIN, GAMMA-2 | 3,82.10^-02^ | -1,60 |
| *GNG7* | GUANINE NUCLEOTIDE-BINDING PROTEIN, GAMMA-7 | 4,40.10^-02^ | -1,08 |
| *GOLT1B* | GOLGI TRANSPORT 1 HOMOLOG B | 1,46.10^-02^ | 0,99 |
| *GPR146* | G-PROTEIN COUPLED RECEPTOR 146 | 2,17.10^-02^ | 1,50 |
| *HLA-DOA* | MAJOR HISTOCOMPATIBILITY COMPLEX, CLASS II, DO ALPHA | 2,30.10^-02^ | -1,67 |
| *IMPAD1* | INOSITOL MONOPHOSPHATASE DOMAIN-CONTAINING PROTEIN 1 | 4,65.10^-02^ | 1,06 |
| *JAZF1* | JUXTAPOSED WITH ANOTHER ZINC FINGER GENE 1 | 4,65.10^-02^ | -1,50 |
| *KYNU* | KYNURENINASE | 2,25.10^-02^ | -1,01 |
| *LYN* | V-YES-1 YAMAGUCHI SARCOMA VIRAL RELATED ONCOGENE HOMOLOG | 4,01.10^-02^ | -0,92 |
| *MACROD2* | MACRO DOMAIN-CONTAINING 2 | 4,01.10^-02^ | -4,16 |
| *MMD* | MONOCYTE-TO-MACROPHAGE DIFFERENTIATION-ASSOCIATED PROTEIN | 6,98.10^-03^ | -1,06 |
| *NAP1L3* | NUCLEOSOME ASSEMBLY PROTEIN 1-LIKE 3 | 4,66.10^-02^ | 1,71 |
| *NEDD4L* | UBIQUITIN PROTEIN LIGASE NEDD4-LIKE | 4,66.10^-02^ | -1,39 |
| *NREP* | NEURONAL REGENERATION RELATED PROTEIN HOMOLOG | 4,66.10^-02^ | -2,99 |
| *OPN3* | OPSIN 3 | 4,66.10^-02^ | -1,40 |
| *PARVG* | PARVIN, GAMMA | 1,93.10^-02^ | -0,92 |
| *PHACTR2* | PHOSPHATASE AND ACTIN REGULATOR 2 | 2,41.10^-02^ | -1,28 |
| *PTEN* | PHOSPHATASE AND TENSIN HOMOLOG | 3,11.10^-02^ | -0,70 |
| *RCAN3* | RCAN FAMILY MEMBER 3 | 2,41.10^-02^ | -3,96 |
| *RGS1* | REGULATOR OF G PROTEIN SIGNALING 1 | 3,11.10^-02^ | 3,15 |
| *SIPA1L1* | SIGNAL-INDUCED PROLIFERATION-ASSOCIATED 1-LIKE PROTEIN 1 | 2,91.10^-02^ | -1,03 |
| *SLC25A37* | SOLUTE CARRIER FAMILY 25, MEMBER 37 | 2,87.10^-02^ | -1,39 |
| *SOCS6* | SUPPRESSOR OF CYTOKINE SIGNALING 6 | 1,46.10^-02^ | 2,31 |
| *SORD* | SORBITOL DEHYDROGENASE | 1,34.10^-02^ | -1,29 |
| *TLE4* | TRANSDUCIN-LIKE ENHANCER OF SPLIT 4 | 4,65.10^-02^ | 0,69 |
| *TM9SF3* | TRANSMEMBRANE 9 SUPERFAMILY MEMBER 3 | 4,99.10^-02^ | 0,81 |
| *TMED8* | TRANSMEMBRANE EMP24DOMAIN CONTAINING 8 | 4,66.10^-02^ | -0,78 |
| *TRIM2* | TRIPARTITE MOTIF-CONTAINING PROTEIN 2 | 3,32.10^-02^ | -3,14 |
| *TSHZ1* | TEASHIRT ZINC FINGER HOMEOBOX 1 | 4,99.10^-02^ | -1,42 |
| *TUBB6* | TUBULIN BETA-6 CHAIN | 4,65.10^-02^ | -2,85 |
| *VCL* | VINCULINE | 2,17.10^-02^ | -1,70 |
| *WIPI1* | WD40 REPEAT PROTEIN INTERACTING WITH PHOSPHOINOSITIDES 1 | 4,86.10^-03^ | 1,18 |

**Table 1 supplemental data: 48 genes identified by the differential analysis.** The fold-changes were established by subtracting the normalized expression values of asymptomatic WM to symptomatic WM. When more than one probe was identified for the same gene, we showed the values of the probe with the best couple p-value/fold-change.
